# Development and validation of a lasso-logistic regression-based risk prediction model for retinopathy in patients with hypertensive disorders of pregnancy

**DOI:** 10.3389/fcvm.2026.1796366

**Published:** 2026-04-15

**Authors:** Shuzhen Gan, Yuanyuan Liu, Caiping Lai, Qiong Li

**Affiliations:** 1 Department of Obstetrics, Ganzhou People’s Hospital, Ganzhou, Jiangxi, China; 2 Department of Obstetrics, Xunwu County People’s Hospital, Ganzhou, Jiangxi, China

**Keywords:** lasso-logistic regression, nomogram, pregnancy induced hypertension, retinopathy, risk prediction model

## Abstract

**Objective:**

To identify risk factors for retinopathy in patients with hypertensive disorders of pregnancy (HDP) and develop a predictive nomogram model.

**Methods:**

A total of 667 patients with PIH treated at our hospital between December 2020 and December 2025 were retrospectively enrolled based on M. Kendall sample size estimation. Patients were randomly assigned to a development cohort (*n* = 400) and an internal validation cohort (*n* = 267) in a 6:4 ratio. According to the occurrence of retinopathy, the modeling group was further divided into a retinopathy group (*n* = 112) and a non-retinopathy group (*n* = 288). Additionally, 200 PIH patients from Xunwu County People's Hospital (January 2021 to December 2024) were included as an external validation cohort. LASSO regression was used to screen potential predictors, followed by multivariate logistic regression to identify independent risk factors. A nomogram prediction model was constructed. Model performance was evaluated using receiver operating characteristic (ROC) curves, calibration plots, and decision curve analysis (DCA).

**Results:**

Among the 400 patients in the modeling group, 112 developed retinopathy, with an incidence of 28.0%. Eight potential predictors were identified by LASSO regression. Multivariate analysis revealed that HDP onset <28 weeks (OR = 7.027), disease duration >3 weeks (OR = 11.548), proteinuria (+++) (OR = 14.535), hematocrit >0.35 (OR = 16.733), and systolic blood pressure (OR = 1.143) were independent risk factors (all *P* < 0.05). Pre-pregnancy BMI (OR = 0.308), albumin (OR = 0.654), and platelet count (OR = 0.961) were independent protective factors (*P* < 0.05). The nomogram demonstrated good calibration in the training, internal validation, and external validation cohorts. The AUC values were 0.948 (95% CI: 0.925–0.970), 0.921 (95% CI: 0.881–0.962), and 0.907 (95% CI: 0.842–0.972), respectively. DCA showed favorable net clinical benefit across a wide range of threshold probabilities.

**Conclusion:**

The nomogram based on clinical and laboratory indicators demonstrated good discrimination and calibration in internal and external validation of retinopathy risk in PIH patients, with good clinical applicability.

## 1 Introduction

Hypertensive disorders of pregnancy are common multisystem diseases in obstetrics, clinically characterized by elevated blood pressure and multi-organ dysfunction, and they are major contributors of maternal morbidity and mortality worldwide (1–4). Hypertensive retinopathy is a common ocular disease, mainly resulting from retinal vascular damage caused by hypertension (5). It not only affects the general hypertensive population but also occurs during pregnancy, posing a serious threat to the health of both mother and fetus (6–8). Therefore, exploring the potential risk factors for retinopathy in patients with hypertensive disorders of pregnancy is of great significance. Prediction models can estimate the probability or risk of specific future outcomes or events in individuals at risk and have been used to identify women at high risk of adverse pregnancy outcomes associated with hypertensive disorders of pregnancy, enabling closer monitoring from early pregnancy and effectively reducing the risk of adverse outcomes (9). In recent years, numerous studies have investigated the influencing factors of retinopathy in patients with hypertensive disorders of pregnancy both domestically and internationally (8); however, few studies have developed and validated quantitative risk prediction models for retinopathy in HDP patients, and the risk attributable to each high-risk factor remains unclear. In accordance with the Transparent Reporting of a multivariable prediction model for Individual Prognosis Or Diagnosis (TRIPOD) guideline, this study aimed to screen for and identify the risk factors for retinopathy in patients with HDP using Lasso-Logistic regression, develop a nomogram model to predict the risk of retinopathy, and comprehensively evaluate its discrimination, calibration, and clinical applicability through internal validation and external validation in an independent cohort, thereby providing an evidence-based basis for early screening and risk stratification of retinopathy in patients with HDP.

### 1.1 Study subjects

Using a convenience sampling method, patients with hypertensive disorders of pregnancy treated at our hospital from December 2020 to December 2025 were selected as the study subjects. The sample size was estimated according to the M. Kendall sample size estimation method, in which the basic sample size was set at 5–10 times the number of included independent variables, with an additional 10% added to account for loss to follow-up. The final sample size for this study was determined to be 667 patients. In the model development cohort, there were 112 retinopathy events, and a total of 8 independent variables were ultimately included in the model. The calculated events per variable (EPV) was 14, meeting the methodological requirement of EPV ≥ 10 for prediction model development. Using a computer-generated random number method, the enrolled patients were randomly assigned in a 6:4 ratio to the model development cohort (*n* = 400) and the internal validation cohort (*n* = 267). In the modeling group, patients were further divided according to the occurrence of retinopathy into an occurrence group (*n* = 112) and a non-occurrence group (*n* = 288). Additionally, 200 patients with hypertensive disorders of pregnancy admitted to Xunwu County People's Hospital from January 2021 to December 2024 were selected as the external validation group.

**Figure 1:**
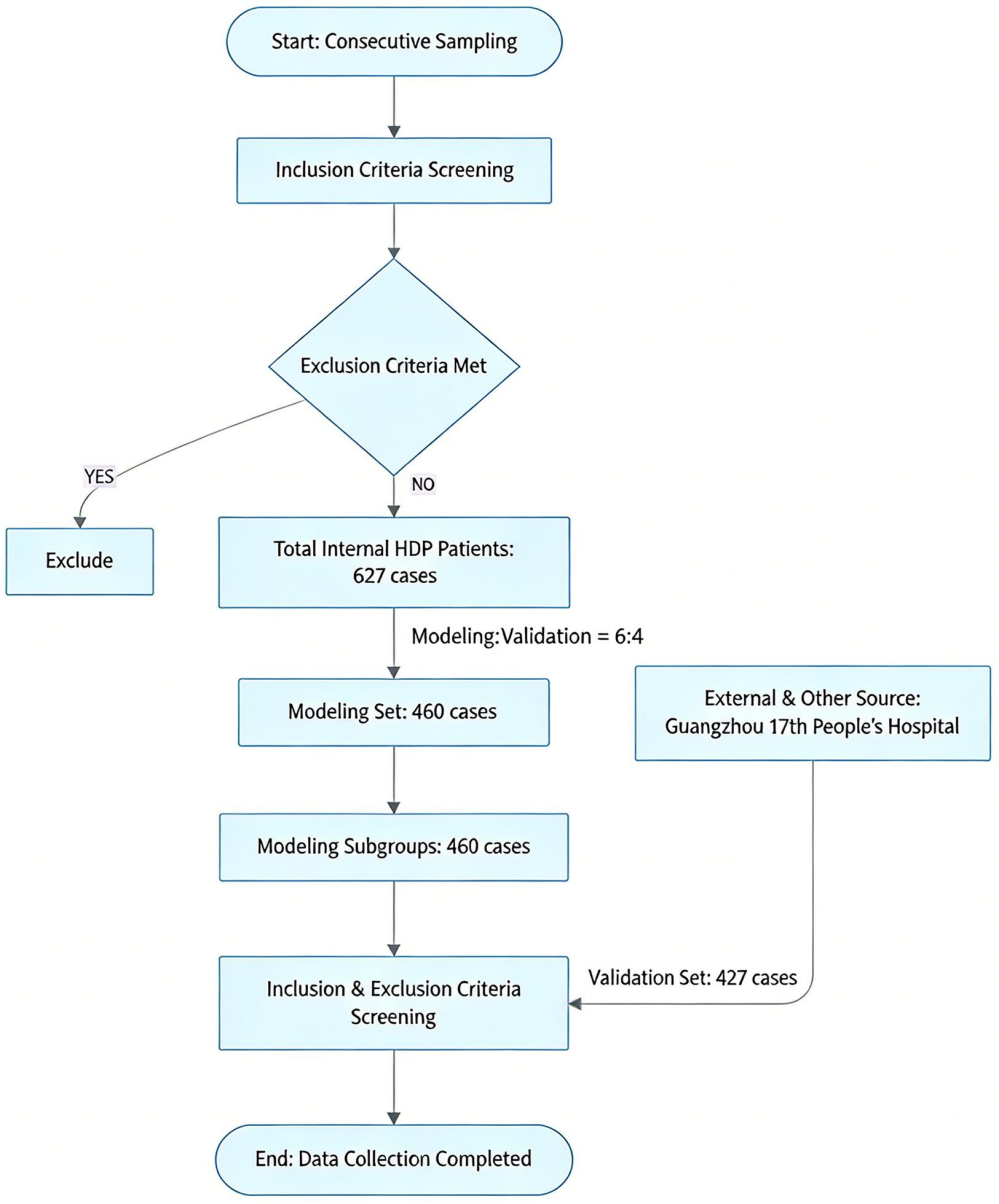
Flowchart of case collection.

Inclusion criteria were as follows: (1) met the diagnostic criteria for hypertensive disorders of pregnancy (10); (2) Met the diagnostic criteria for retinopathy (11). In this study, the diagnosis and grading of retinopathy in patients with hypertensive disorders of pregnancy were independently assessed in a blinded manner by two senior attending ophthalmologists using direct ophthalmoscopy combined with color fundus photography; (3) age >18 years; (4) singleton pregnancy. Exclusion criteria were as follows: (1) presence of diabetes, inflammatory or immune diseases, or neurological diseases; (2) renal or hepatic dysfunction or coagulation disorders; (3) pre-existing hypertension or ocular diseases; (4) psychiatric disorders. The study flowchart is shown in Figure 1.

### 1.2 Data collection methods

Based on literature review and expert evaluation, a total of 16 risk factors were included in this study. A self-designed baseline data questionnaire was used to collect patient information, including age, gestational age, pre-pregnancy body mass index (BMI), disease type, gravidity and parity, edema, time of onset of hypertensive disorders of pregnancy, disease duration, systolic blood pressure, and diastolic blood pressure. At 5:00 a.m. on the morning of the second day after admission, 2 mL of fasting venous blood was collected and placed in a heparin anticoagulant tube, gently inverted five times to mix. A Beckman Coulter hematology analyzer (COULTER LH 750) was used to measure routine blood parameters, including proteinuria, hematocrit, albumin (ALB), platelets (PLT), alanine aminotransferase (ALT), and aspartate aminotransferase (AST).

### 1.3 Statistical methods

Study data were analyzed using SPSS 22.0 software. Continuous variables were expressed as mean ± standard deviation, and categorical variables were expressed as number/percentage. Intergroup comparisons were performed using the *t* test (for normally distributed data) and the chi-square test. Before performing LASSO regression analysis, collinearity diagnostics were conducted for the 16 candidate predictor variables using the variance inflation factor (VIF) method (VIF < 10), and the linearity of continuous variables with the logit was assessed using the Box–Tidwell test (*P* > 0.05); all results met the requirements for analysis. The comparability of baseline characteristics among the three groups was evaluated using standardized differences (SD) in combination with *P* values (SD < 0.1 indicating balance), thereby ensuring the absence of grouping bias. After univariate analysis, potential risk factors were screened by LASSO regression to eliminate redundancy, and independent risk factors were subsequently identified using Logistic regression. A nomogram prediction model was constructed using the rms package in R software. Internal and external validation of the model were performed, and the area under the curve (AUC) was calculated to evaluate the discriminative ability of the model. The Hosmer–Lemeshow test and calibration curves were used to assess the consistency of the nomogram prediction model, and decision curve analysis (Decision Curve Analysis, DCA) was employed to evaluate its clinical utility. A *P* value <0.05 was considered statistically significant.

## 2 Results

### 2.1 Comparison of baseline characteristics among the three groups

The results of baseline characteristic comparisons among the groups are shown in Table 1. Baseline comparability between groups was evaluated using standardized differences (SD) in combination with *P* values, with SD < 0.1 considered indicative of good balance. The results showed that the standardized differences for all baseline characteristics between the groups were <0.1 (all *P* > 0.05), indicating that the baseline characteristics of the two groups were highly balanced, with no obvious bias.

**Table 1:** Comparison of baseline characteristics among the modeling group and validation groups [*n* (%)/(±)].

Variable	Modeling group (*n* = 400)	Internal Validation group (*n* = 267)	External validation group (*n* = 200)	*F*/*χ*^2^	*P* value	Standardized difference (SD)
Disease type						
Chronic hypertension with preeclampsia	52 (13.00)	40 (17.94)	32 (16.00)	4.601	0.596	0.062
Eclampsia	63 (15.75)	33 (14.80)	28 (14.00)			
Gestational hypertension	152 (38.00)	90 (40.36)	76 (38.00)			
Preeclampsia	133 (33.25)	60 (26.91)	64 (32.00)			
Time of onset of hypertensive disorders of pregnancy (weeks)						
<28	151 (37.75)	89 (33.33)	72 (36.00)	1.356	0.508	0.045
≥28	249 (62.25)	178 (66.67)	128 (64.00)			
Disease duration (weeks)						
≤3	246 (61.50)	152 (56.93)	124 (62.00)	1.297	0.523	0.041
>3	154 (38.50)	115 (43.07)	76 (38.00)			
Gravidity and parity						
<2	373 (93.25)	239 (89.51)	186 (93.00)	3.379	0.185	0.058
≥2	27 (6.75)	28 (10.49)	14 (7.00)			
Proteinuria						
+	161 (40.25)	98 (36.70)	78 (39.00)	1.965	0.923	0.032
++	98 (24.50)	62 (23.22)	46 (23.00)			
+++	81 (20.25)	63 (23.60)	42 (21.00)			
++++	60 (15.00)	44 (16.48)	34 (17.00)			
Hematocrit (L/L)						
≤0.35	235 (58.75)	167 (62.55)	118 (59.00)	1.065	0.587	0.038
>0.35	165 (41.25)	100 (37.45)	82 (41.00)			
Edema						
Yes	324 (81.00)	207 (77.53)	158 (79.00)	1.218	0.544	0.043
No	76 (19.00)	60 (22.47)	42 (21.00)			
Age (years)	30.45 ± 3.48	30.92 ± 3.35	30.63 ± 3.51	1.468	0.227	0.051
Gestational age (weeks)	33.15 ± 1.22	33.05 ± 1.35	32.97 ± 1.42	1.347	0.260	0.047
Pre-pregnancy BMI (kg/m^2^)	20.18 ± 1.66	20.36 ± 1.52	20.25 ± 1.58	1.014	0.363	0.036
ALB (g/L)	24.36 ± 3.61	23.94 ± 3.55	24.12 ± 3.67	1.117	0.328	0.039
ALT (U/L)	37.52 ± 6.65	37.13 ± 6.46	36.89 ± 6.58	0.681	0.506	0.029
AST (U/L)	20.98 ± 3.44	20.44 ± 3.59	20.71 ± 3.62	1.893	0.151	0.055
Systolic blood pressure (mmHg)	148.58 ± 14.35	146.86 ± 15.23	147.65 ± 14.89	1.109	0.330	0.040
Diastolic blood pressure (mmHg)	96.88 ± 10.07	97.02 ± 10.01	96.53 ± 10.15	0.141	0.869	0.018
PLT (×10⁹/L)	148.02 ± 29.61	148.57 ± 28.43	147.89 ± 29.17	0.040	0.961	0.012

### 2.2 Univariate analysis of retinopathy in the modeling group

According to statistical analysis, among the 400 patients with hypertensive disorders of pregnancy in the modeling group, 112 developed retinopathy, with an incidence rate of 28.00%. The results of univariate analysis showed that there were statistically significant differences between the occurrence group and the non-occurrence group in terms of time of onset of hypertensive disorders of pregnancy, disease duration, proteinuria, hematocrit, pre-pregnancy BMI, ALB, systolic blood pressure, diastolic blood pressure, and PLT (*P* < 0.05). There were no statistically significant differences between the two groups in disease type, gravidity and parity, edema, age, gestational age, ALT, or AST (*P* > 0.05). See Table 2.

**Table 2:** Univariate analysis of retinopathy in the modeling group [*n* (%)/(±)].

Factors	Modeling group		*F*/*t*/*χ^2^*value	*P* value
	Occurrence group (*n* = 112)	Non-occurrence group (*n* = 288)		
Disease type				
Chronic hypertension with preeclampsia	16 (14.29)	36 (12.50)	0.418	0.936
Eclampsia	16 (14.29)	47 (16.32)		
Gestational hypertension	43 (38.38)	109 (37.85)		
Preeclampsia	37 (33.04)	96 (33.33)		
Time of onset of hypertensive disorders of pregnancy (weeks)				
<28	77 (68.75)	74 (25.69)	63.614	0.000
≥28	35 (31.25)	214 (74.31)		
Disease duration (weeks)				
≤3	28 (25.00)	218 (75.69)	87.526	0.000
>3	84 (75.00)	70 (24.31)		
Gravidity and parity				
<2	102 (91.09)	271 (94.10)	1.173	0.279
≥2	10 (8.93)	17 (5.90)		
Proteinuria				
+	11 (9.82)	150 (52.08)	102.339	0.000
++	22 (19.64)	76 (26.39)		
+++	37 (33.04)	44 (15.28)		
++++	42 (37.50)	18 (6.25)		
Hematocrit (L/L)				
≤0.35	17 (15.18)	218 (75.69)	121.859	0.000
>0.35	95 (84.82)	70 (24.31)		
Edema				
Yes	91 (81.25)	233 (80.90)	0.006	0.937
No	21 (18.75)	55 (19.10)		
Age (years)	30.45 ± 3.48	30.90 ± 3.60	1.133	0.258
Gestational age (weeks)	33.15 ± 1.22	33.38 ± 1.32	1.598	0.111
Pre-pregnancy BMI (kg/m^2^)	20.18 ± 1.66	23.39 ± 1.40	19.515	0.000
ALB (g/L)	24.36 ± 3.61	32.55 ± 4.80	16.344	0.000
ALT (U/L)	37.52 ± 6.65	36.44 ± 6.41	1.497	0.135
AST (U/L)	20.98 ± 3.44	21.35 ± 3.89	0.881	0.379
Systolic blood pressure (mmHg)	148.58 ± 14.35	123.22 ± 10.60	19.354	0.000
Diastolic blood pressure (mmHg)	96.88 ± 10.07	84.25 ± 8.44	12.708	0.000
PLT (×10⁹/L)	148.02 ± 29.61	174.33 ± 35.20	7.004	0.000

### 2.3 Multivariable lasso-logistic regression analysis of retinopathy in patients with hypertensive disorders of pregnancy

**Figure 2:**
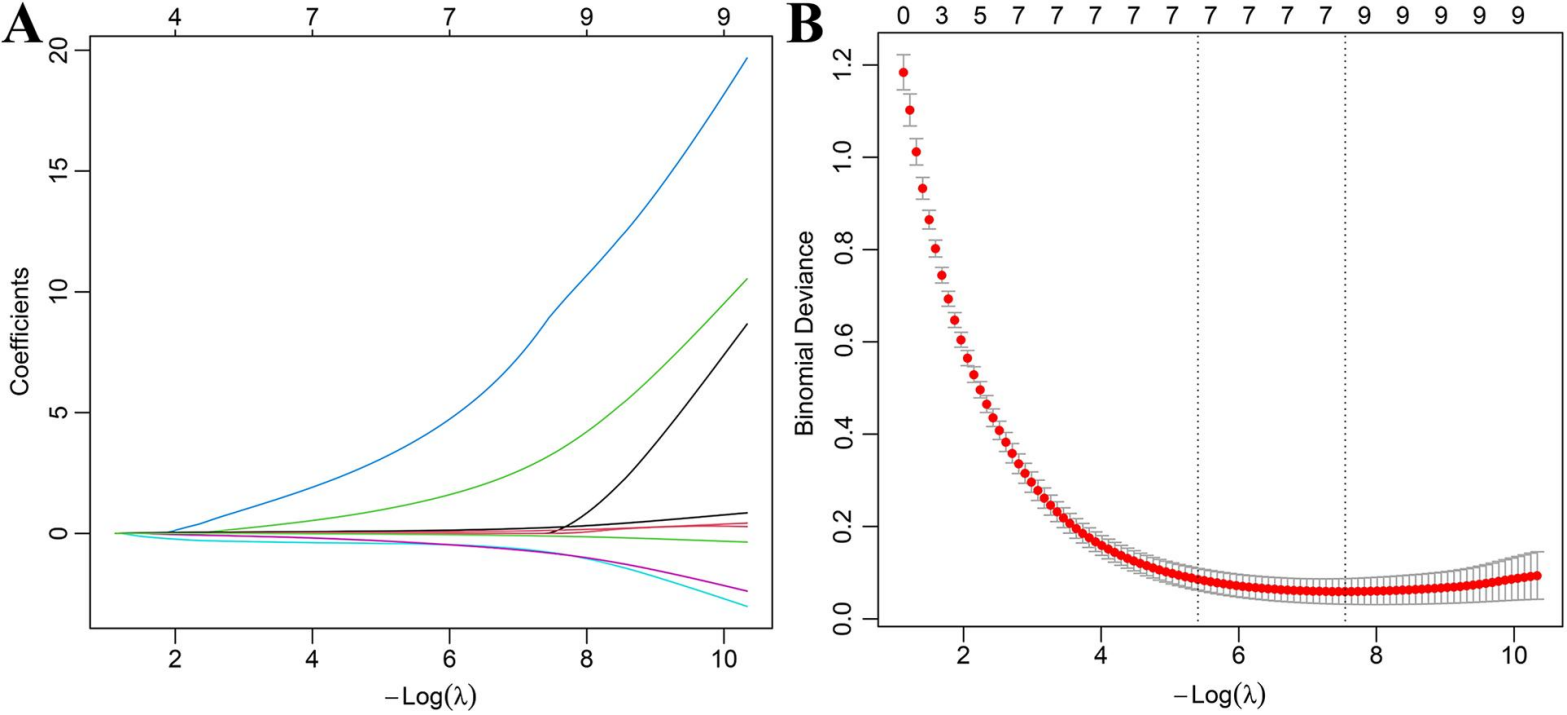
LASSO-logistic regression analysis for retinopathy in hypertensive disorders of pregnancy **(A)** cross-validation for selection of *λ*; **(B)** LASSO coefficient profiles.

In the modeling group, the occurrence of retinopathy in patients with hypertensive disorders of pregnancy was used as the dependent variable (no = 0, yes = 1), and the variables with significant differences in Table 2 were used as independent variables (a total of 9). Before performing LASSO regression analysis, collinearity diagnostics were conducted for the 16 initially included candidate predictor variables using the variance inflation factor (VIF) method, with VIF <10 indicating no significant collinearity, while the linear relationship of continuous variables in the logit was assessed using the Box–Tidwell test, with *P* > 0.05 indicating compliance; all results met the requirements for analysis. On this basis, Lasso analysis was performed using R software. The results showed that when the penalty coefficient *λ*min = 0.0044857, the model demonstrated good performance with the fewest influencing factors, and a total of 8 predictive factors were finally selected (see Figure 2).

With retinopathy occurrence as the dependent variable (no = 0, yes = 1), predictive factors such as time of onset of hypertensive and disorders of pregnancy were sequentially included in the logistic model. The variable assignment methods are shown in Table 3. The results showed that time of onset of hypertensive disorders of pregnancy <28 weeks (OR = 7.027, 95% CI: 1.310–37.707), disease duration >3 weeks (OR = 11.548, 95% CI: 1.717–77.671), proteinuria (++++) (OR = 14.535, 95% CI: 1.443–146.393), hematocrit >0.35 (OR = 16.733, 95% CI: 2.224–125.907), and systolic blood pressure (OR = 1.143, 95% CI: 1.065–1.226) were risk factors for retinopathy (*P* < 0.05). Pre-pregnancy BMI (OR = 0.308, 95% CI: 0.167–0.570), ALB (OR = 0.654, 95% CI: 0.501–0.854), and PLT (OR = 0.961, 95% CI: 0.931–0.991) were protective factors for retinopathy (*P* < 0.05). See Table 4.

**Table 3:** Variable assignment methods.

Variable	Assignment method
Time of onset of hypertensive disorders of pregnancy (weeks)	<28 = 1, ≥28 = 0
Disease duration (weeks)	≤3 = 0, >3 = 1
Proteinuria	+ = 0, ++ = 1, +++ = 2, ++++ = 3
Hematocrit (L/L)	>0.35 = 1, ≤0.35 = 0
Pre-pregnancy BMI (kg/m^2^)	Continuous variable
ALB (g/L)	Continuous variable
Systolic blood pressure (mmHg)	Continuous variable
Diastolic blood pressure (mmHg)	Continuous variable
PLT (×10⁹/L)	Continuous variable

**Table 4:** Multivariable logistic regression analysis of risk factors for retinopathy in patients with hypertensive disorders of pregnancy.

Variable	*B*	SE	Wald	*P*	OR	95% CI	
Time of onset of hypertensive disorders of pregnancy	1.950	0.857	5.174	0.023	7.027	1.310	37.707
Disease duration	2.447	0.972	6.329	0.012	11.548	1.717	77.671
Hematocrit	2.817	1.030	7.486	0.006	16.733	2.224	125.907
Pre-pregnancy BMI	−1.176	0.314	14.066	0.000	0.308	0.167	0.570
ALB	−0.424	0.136	9.754	0.002	0.654	0.501	0.854
Systolic blood pressure	0.134	0.036	13.879	0.000	1.143	1.065	1.226
PLT	−0.040	0.016	6.533	0.011	0.961	0.931	0.991
Proteinuria (++++)	2.677	1.178	5.159	0.023	14.535	1.443	146.393

### 2.4 Construction of the prediction model

**Figure 3:**
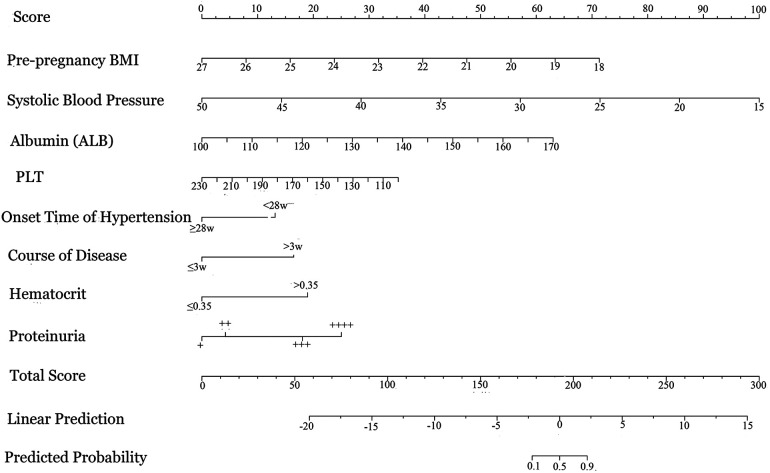
Nomogram for predicting retinopathy in hypertensive disorders of pregnancy.

A nomogram prediction model was constructed using the important predictive factors identified in the Logistic regression analysis. The results showed that for every 1 kg/m^2^ decrease in pre-pregnancy BMI, the nomogram score increased by 28 points; for every 5 g/L decrease in ALB, the nomogram score increased by 50 points; for every 10 mmHg increase in systolic blood pressure, the nomogram score increased by 33 points; for every 20 × 10⁹/L decrease in PLT, the nomogram score increased by 21 points; proteinuria ++++ increased the nomogram score by 78 points; time of onset of hypertensive disorders of pregnancy <28 weeks increased the weight by 13 points; disease duration >3 weeks increased the score by 43 points; and hematocrit >0.35 increased the score by 55 points. When the total nomogram score for a patient with hypertensive disorders of pregnancy was 206 points, the risk of retinopathy was 0.9. See Figure 3.

### 2.5 Evaluation of the prediction model for retinopathy in patients with hypertensive disorders of pregnancy

**Figure 4:**
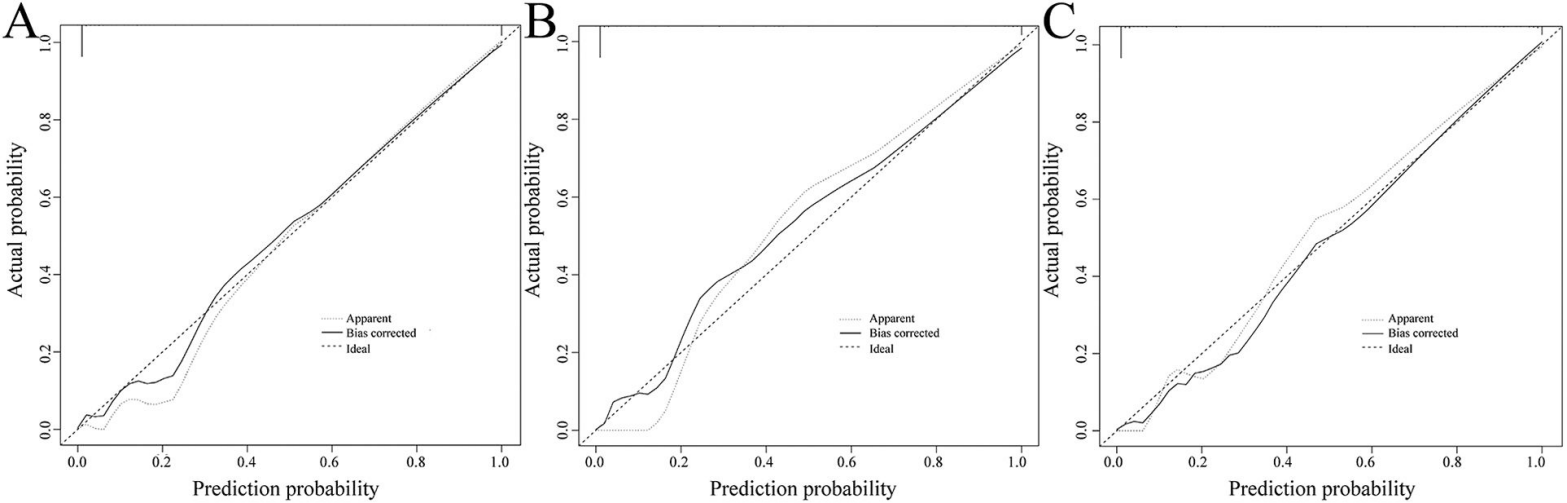
ROC curves of the prediction model **(A)** training cohort; **(B)** internal validation cohort; **(C)** external validation cohort.

**Figure 5:**
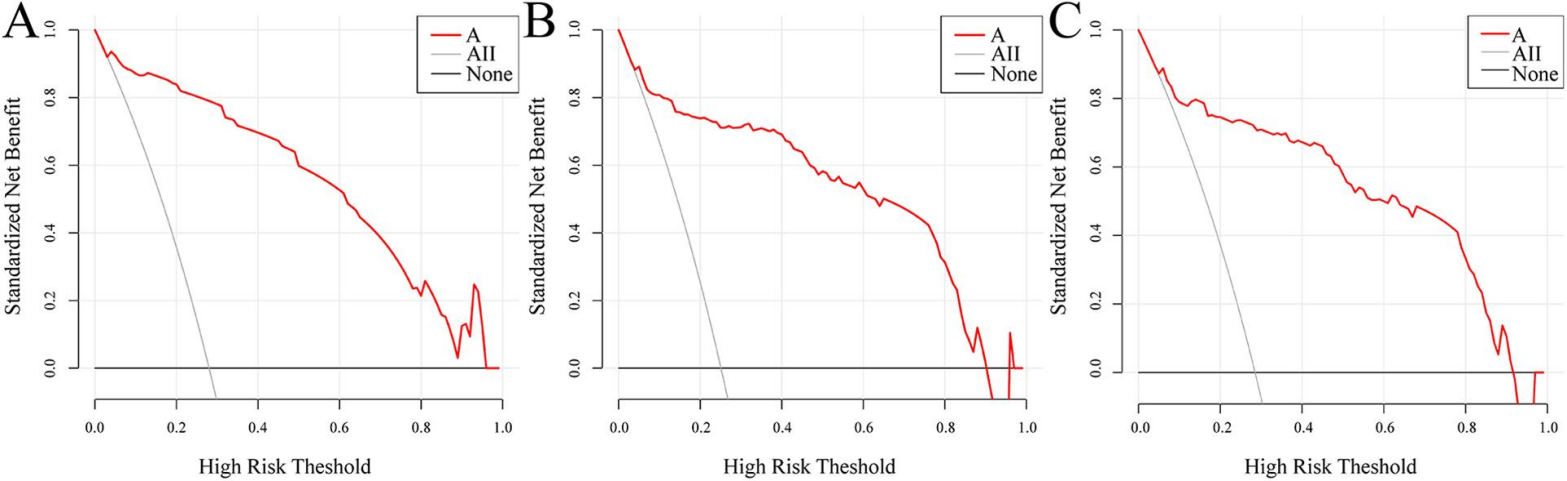
Hosmer–Lemeshow goodness-of-fit test. **(A)** Calibration curve of the modeling group; **(B)** calibration curve of the internal validation group; **(C)** Calibration curve of the external validation group.

The ROC curve results showed that the areas under the curve for predicting retinopathy in the modeling group, internal validation group, and external validation group were 0.948 (95% CI: 0.925–0.970), 0.921 (95% CI: 0.881–0.962), and 0.907 (95% CI: 0.842–0.972), respectively, and the Youden indices were 0.805, 0.751, and 0.730, respectively, with optimal cutoff values of 0.29, 0.41, and 0.37, respectively, indicating high predictive value of the model. See Figure 4. The Hosmer–Lemeshow goodness-of-fit test showed that *χ*^2^ = 8.002 and *P* = 0.433 in the modeling group, *χ*^2^ = 7.910 and *P* = 0.442 in the internal validation group, and *χ*^2^ = 7.838 and *P* = 0.128 in the external validation group. These results indicated no obvious calibration bias in the model, and good agreement between the predicted and actual risks of retinopathy. The calibration curves showed a good fit between the corrected curve and the ideal curve, further confirming the good calibration performance of the model. See Figure 5.

### 2.6 Decision curve analysis

**Figure 6:**
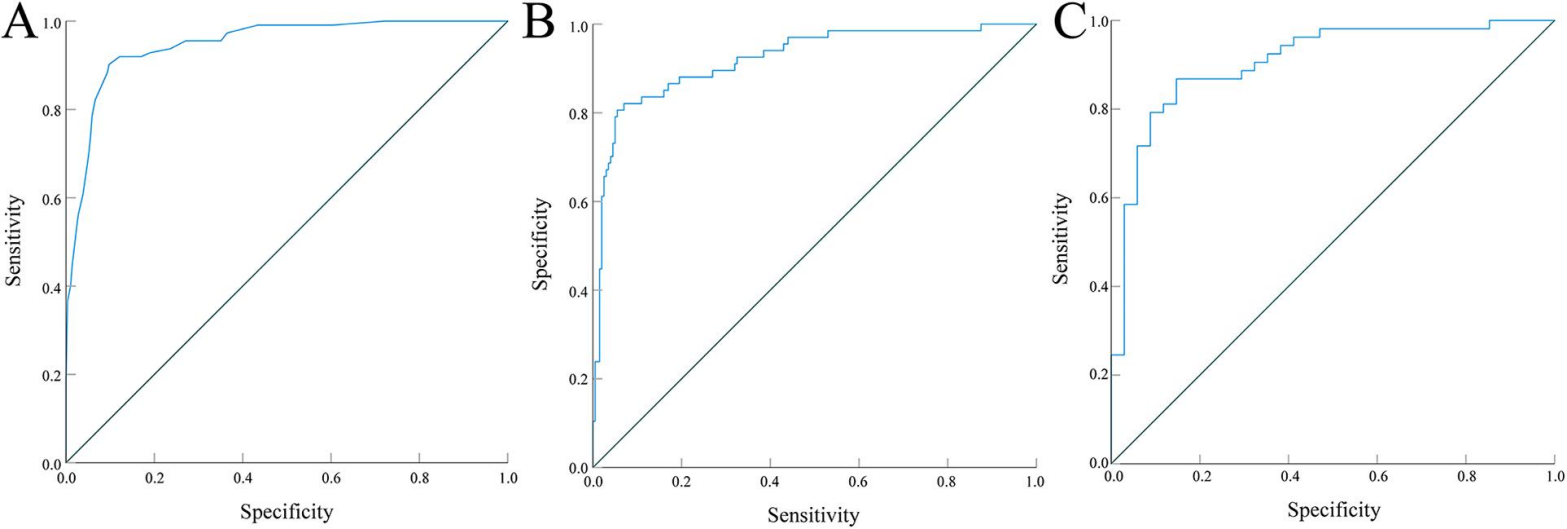
Decision curve analysis of the prediction model. **(A)** Training cohort; **(B)** internal validation cohort; **(C)** external validation cohort.

The DCA curves showed that within the threshold probability range of 0.05–0.85, the net benefit of the nomogram model constructed in this study was higher than that of both the “treat-all” and “treat-none” strategies in the modeling group (Figure 6A), the internal validation group (Figure 6B), and the external validation group (Figure 6C). This indicates that the model has good practical value in clinical settings and can assist clinicians in conducting individualized risk assessment and decision-making. See Figure 6.

## 3 Discussion

Retinopathy is a common complication in patients with hypertensive disorders of pregnancy (12, 13). Studies have shown that 25%–50% of patients with hypertensive disorders of pregnancy experience involvement of the visual system, mainly manifested as narrowing of retinal arterioles, and may also present with retinal edema, exudation, cotton-wool spots, hemorrhage, or even retinal detachment (14, 15). This complication not only causes irreversible damage to maternal visual function but may also indirectly threaten normal fetal development by affecting placental blood circulation. Therefore, an in-depth exploration of its pathogenesis and risk factors is of great clinical significance for improving maternal and fetal outcomes.

In this study, the incidence of retinopathy in patients with hypertensive disorders of pregnancy was 28.00%, which is basically consistent with the findings of Ye et al. (16) (27.8%). This indicates that this complication has a relatively high incidence in patients with hypertensive disorders of pregnancy and warrants close clinical attention. Multivariate analysis showed that time of onset of hypertensive disorders of pregnancy <28 weeks was an important risk factor (OR = 7.027). This may be because early pregnancy is a critical period for fetal organ development and placental formation. The occurrence of hypertension during this period can directly interfere with normal placental vascular remodeling, leading to systemic vascular endothelial injury in the mother and subsequently affecting the stability of retinal vessels (17). In addition, elevated blood pressure in early pregnancy may further aggravate vasospasm and increased vascular permeability by activating the renin–angiotensin–aldosterone system, thereby accelerating the development of retinopathy (18). Therefore, for patients who develop hypertension in early pregnancy, fundus examination should be performed as early as possible, and blood pressure monitoring and intervention should be strengthened. Disease duration >3 weeks (OR = 11.548) was also an important risk factor for retinopathy. With prolonged disease duration, persistently elevated blood pressure causes retinal arterioles to remain in a long-term spastic state, leading to repeated endothelial cell injury and increased vascular wall permeability, resulting in pathological changes such as edema and exudation. Meanwhile, long-term hypertension can also cause retinal vascular sclerosis and insufficient blood perfusion, leading to retinal ischemia and hypoxia and aggravating the severity of lesions (19). This finding suggests that clinicians should pay close attention to disease course management in patients with hypertensive disorders of pregnancy. For patients with a disease duration exceeding 3 weeks, treatment regimens should be adjusted in a timely manner to maintain blood pressure within an ideal range, thereby reducing the risk of retinopathy. Proteinuria (++++) (OR = 14.535) and hematocrit >0.35 (OR = 16.733) were the two risk factors with relatively high OR values in this study. Proteinuria is an important indicator reflecting renal vascular endothelial injury, and the vascular structures of the kidney and retina are similar, both being terminal microvascular beds. Therefore, renal injury often indicates extensive involvement of the systemic microvascular system (20, 21). Massive proteinuria (++++) indicates severe endothelial damage and significantly increased permeability, a pathological process that also occurs in retinal vessels, leading to retinal edema and exudation (22). Among these factors, hematocrit >0.35 was the risk factor most strongly associated with retinopathy in this study. Its underlying mechanism may be closely related to the systemic pathophysiological changes of hypertensive disorders of pregnancy and the regulatory characteristics of ocular microcirculation. The core pathological change in hypertensive disorders of pregnancy is systemic arteriolar spasm, whereas hematocrit >0.35 indicates a state of hemoconcentration. Primarily, hemoconcentration increases blood viscosity, which—synergistically with retinal arteriolar spasms—slows microcirculatory flow, leading to abnormal retinal capillary perfusion pressure and insufficient blood perfusion, causing retinal tissue ischemia and hypoxia, while also increasing the risk of intravascular microthrombus formation and disrupting the stability of the blood supply (23). On the other hand, the elevated perfusion pressure induced by hemoconcentration may exceed the autoregulatory threshold of the retinal capillaries, impair endothelial cell junctions and the integrity of the blood–retinal barrier, and lead to plasma leakage, thereby inducing retinal edema and exudation. In addition, hemoconcentration reduces plasma colloid osmotic pressure, which may further aggravate retinal fluid retention and accelerate disease progression. Furthermore, hemoconcentration may worsen choroidal microcirculatory stasis, resulting in insufficient blood supply to the retinal pigment epithelium and loss of its nutritional and barrier-protective functions for the retina, thereby creating a vicious cycle of “choroidal–retinal” microcirculatory dysfunction and ultimately promoting the occurrence and progression of retinopathy (24). Elevated systolic blood pressure (OR = 1.143) was also identified as a risk factor for retinopathy. Systolic blood pressure is an important indicator reflecting vascular contractile function. Increased systolic blood pressure directly raises the pressure load on retinal vessels, leading to endothelial cell injury and reduced vascular wall elasticity (25). Long-term high pressure can cause narrowing and sclerosis of retinal arterioles and even arteriovenous crossing changes, which are typical pathological manifestations of hypertensive retinopathy (26). Compared with diastolic blood pressure, systolic blood pressure has a more pronounced damaging effect on retinal vessels, which may be related to its higher peak pressure and stronger impact on the vascular wall (27). Therefore, clinical treatment should focus on controlling systolic blood pressure and maintaining it below 140 mmHg to reduce pressure-related damage to retinal vessels. This study also found that pre-pregnancy BMI (OR = 0.308), ALB (OR = 0.654), and PLT (OR = 0.961) were protective factors against retinopathy. Obesity is generally considered in clinical practice to be closely associated with endothelial dysfunction, insulin resistance, and hemodynamic abnormalities, and is regarded as a risk factor for hypertension and vascular complications (28). However, the unexpected finding regarding BMI does not negate the link between obesity and vascular dysfunction; rather, it may reflect specific cohort characteristics. Specifically, the overall pre-pregnancy BMI of the included participants was within the normal to relatively low range (20.18 ± 1.66 kg/m^2^ in the model development group), and no severely obese population was included, which may have led to sample bias. ALB is an important substance for maintaining vascular oncotic pressure. Adequate ALB levels can reduce intravascular fluid leakage and alleviate retinal edema (29). In addition, ALB has antioxidant and anti-inflammatory properties, which can mitigate vascular endothelial injury (30). PLT participates in vascular repair and hemostasis, and normal PLT levels can promote healing of damaged vascular endothelium and reduce hemorrhage and exudation (31). These findings suggest that while focusing on risk factors, clinicians should also emphasize interventions targeting protective factors, such as guiding patients to reasonably control body weight, supplement high-quality protein, and monitor platelet levels, in order to reduce the risk of retinopathy.

Regarding model validation, the nomogram model constructed in this study achieved AUC values of 0.948, 0.921, and 0.907 in the modeling group, internal validation group, and external validation group, respectively, indicating good discriminative ability. The calibration curves showed good agreement between the predicted and ideal curves, suggesting that the predicted values were largely consistent with the actual risk of occurrence. DCA demonstrated that within the threshold probability range of 0.05–0.85, the net benefit of the model was consistently higher than that of the “treat-all” and “treat-none” strategies, uggesting the model's high clinical utility, although its routine application remains subject to further prospective validation.

In summary, the nomogram model constructed in this study based on factors such as time of onset of hypertensive disorders of pregnancy, disease duration, proteinuria, hematocrit, systolic blood pressure, pre-pregnancy BMI, ALB, and PLT demonstrated good discrimination and calibration in both the internal and external validation cohorts, indicating good clinical practicability. This model facilitates early risk assessment and the identification of high-risk patients, enabling the formulation of individualized interventions, such as enhanced blood pressure monitoring, regular fundus examinations, supplementation with high-quality protein, and weight control, to reduce the risk of retinopathy and improve maternal and fetal outcomes. This study also has certain limitations. Convenience sampling was used to select the study participants. Although this method is simple to implement and efficient for enrollment, and can rapidly meet the sample size requirements for model development, it may introduce selection bias. Specifically, as the sample was limited to two hospitals, geographic and institutional factors—such as medical resource accessibility—might affect the representativeness of the cohort. This may limit the generalizability of the model, and prospective multicenter validation is still required before its routine clinical application.

## Data Availability

The original contributions presented in the study are included in the article/Supplementary Material, further inquiries can be directed to the corresponding author.
